# Grape Stalks as a Sustainable Feed Supplement for Dairy Cows: A Preliminary In Vivo Study on Milk Microbiota and Cheese Quality

**DOI:** 10.3390/ani16030388

**Published:** 2026-01-26

**Authors:** Giulia Dallavalle, Giorgia Secchi, Andrea Mancini, Nicola Cologna, Urska Vrhovsek, Andrea Angeli, Eugenio Aprea, Jessica Zambanini, Pavel Solovyev, Luana Bontempo, Emanuela Betta, Franco Biasioli, Thomas Zanon, Elena Franciosi

**Affiliations:** 1 Research and Innovation Centre, Fondazione Edmund Mach (FEM), 38098 San Michele all’Adige, Italy; giulia.dallavalle.3@studenti.unipd.it (G.D.); giorgia.secchi@fmach.it (G.S.); andrea.mancini@fmach.it (A.M.); urska.vrhovsek@fmach.it (U.V.); andrea.angeli@fmach.it (A.A.); pavel.solovyev@fmach.it (P.S.); luana.bontempo@fmach.it (L.B.); emanuela.betta@fmach.it (E.B.); franco.biasioli@fmach.it (F.B.); 2 Department of Industrial Engineering, University of Padova, 35131 Padova, Italy; 3 ONFoods—Research and Innovation Network on Food and Nutrition Sustainability, Safety and Security—Working on Foods, 43121 Parma, Italy; 4 Trentingrana Consorzio dei Caseifici Sociali Trentini s.c.a., 38121 Trento, Italy; cologna@concast.tn.it; 5 Center Agriculture Food Environment, University of Trento, 38098 San Michele all’Adige, Italy; eugenio.aprea@unitn.it; 6 Faculty of Agricultural, Environmental and Food Sciences, Free University of Bolzano, 39100 Bolzano, Italy; thomas.zanon@unibz.it

**Keywords:** circular economy, grape stalk, cheese making, milk quality, dairycow, microbiome, NMR, VOCs

## Abstract

In the Trentino Alto Adige region, disposal of grape stalks (GS) represents a major cost for wineries, despite their content of phenolic and tannin-rich compounds with potential functional value in ruminant nutrition. This study evaluated whether dietary GS supplementation could influence milk microbiota and cheese quality, supporting local circular-economy strategies. GS from three red cultivars (L-GS, CS-GS, M-GS) were dried, milled, and assessed for safety; their average total polyphenol content was approximately 15 g/kg DM. 3 Holstein cows underwent a 7-week trial consisting of alternating control (CTRL) and GS-supplemented periods (2% DM). Weekly milk samples (*n* = 21) and corresponding mini-cheeses (*n* = 21) were analyzed. GS supplementation did not affect milk coliforms (2.3–2.5 log CFU/mL), while total mesophilic counts were significantly lower in M-GS (2.8 ± 0.46 log CFU/mL) than in CTRL (4.5 ± 0.71; *p* < 0.05). Acinetobacter dominated the milk microbiota but decreased from 34.0% in CTRL to 18.0% in L-GS. Cheese total polyphenols were highest in CS-GS (224 ± 34 mg/kg). ^1^H-NMR and VOCs profiling indicated cultivar-dependent shifts in carbohydrate-related metabolites and short-chain fatty acids. Overall, GS supplementation subtly modulated cheese biochemical and aromatic traits without impairing technological performance, supporting the valorization of winery by-products within integrated dairy-viticulture systems.

## Simple Summary

Grape stalks are the woody stems that remain after grapes are processed for winemaking. In the Trentino-Alto Adige region, large amounts of these materials are produced every year, and their disposal represents a cost for wineries. Instead of treating grape stalks as waste, they could be used as a valuable resource for dairy farms. In this study, we explored whether adding a small amount of grape stalks to the daily diet of dairy cows could influence milk composition and the cheese made from that milk. A seven-week alternating design was applied to three cows, which were fed either their usual diet or the same diet supplemented with grape stalks in alternating weeks. Milk was collected weekly, and small cheeses were produced to examine possible changes in quality, bacterial composition, and aroma. We found that feeding grape stalks did not negatively affect milk quality or safety. Some natural bacterial groups in the milk decreased when cows received grape stalks, and the cheeses showed small but consistent differences in natural plant compounds and in smell and flavor. These preliminary findings suggest that grape stalks could become a valuable and sustainable resource for dairy farms, supporting local circular economy practices and reducing waste from winemaking.

## 1. Introduction

In the context of the ongoing environmental crisis and the increasing pressure on natural resources, the circular bio-economy model has emerged as a crucial paradigm for sustainable development in the agri-food sector [1]. This approach aims to reduce waste and enhance the value of biological materials by reintegrating them into productive chains, thereby promoting more efficient and regenerative resource use [2]. The transition from linear production models (produce–consume–dispose) to circular systems requires identifying innovative strategies for valorizing agricultural by-products, especially those generated in large volumes by local supply chains [3].

In Trentino-South Tyrol, a mountainous region in Northern Italy, agriculture plays a fundamental role not only in food production but also in landscape preservation and cultural identity. The region is characterized by multifunctional farming systems, where wine production often coexists with animal husbandry, particularly dairy farming [4]. While this integration creates opportunities for circular practices, it also highlights logistical and economic challenges in managing agro-industrial residues.

One of the main waste products generated by local wineries is grape stalks (GS), the lignified parts of the grape cluster that are separated during destemming. GS represents up to 7% of the grape weight and, despite being rich in fibrous and bioactive compounds, is still largely considered as a waste. Their disposal, commonly via composting or biomass incineration [5], is associated with costs, environmental concerns, and increasing pressure from producers for alternative, value-generating uses. In recent years, winemakers have shown growing interest in finding more sustainable disposal solutions for GS, especially amid evolving regulations and climate-related constraints. At the same time, dairy farmers in the region are increasingly receptive to feed innovations that support animal health, enhance product quality, and promote local resource circularity [6]. This context opens the way for cross-sectoral strategies that connect wine and dairy production through the functional use of by-products.

Scientific interest in grape-derived materials as feed ingredients has been growing, particularly in grape pomace, seeds, and skins [7,8,9]. These materials are rich in polyphenols, including tannins, flavonoids, and phenolic acids, which are known for their antioxidant, antimicrobial, and anti-inflammatory activities [10]. Polyphenols can modulate ruminal fermentation by affecting microbial populations, volatile fatty acid (VFA) production, and nitrogen metabolism. In vitro studies have shown that polyphenol-rich grape residues can reduce ammonia levels, alter the acetate-to-propionate ratio, and potentially decrease enteric methane production [8,11].

While most research has focused on grape pomace, recent work suggests that grape stalks, although less explored, contain polyphenols comparable to those in pomace [12], including condensed tannins [13]. Moreover, only a limited number of studies have evaluated in vivo the impact of grape polyphenols on rumen function and dairy product composition [14,15]. Although the rumen is a highly degradative environment, some polyphenols or their transformation products may resist microbial breakdown and reach the bloodstream, potentially affecting mammary metabolism or transferring to milk [16]. Recent analytical advances, including NMR-based metabolomics, have enabled more precise detection of such compounds in complex matrices like milk and cheese [17]. This raises questions about how grape-derived dietary polyphenols may influence not only rumen microbiota but also the nutritional and functional properties of dairy products.

In this context, the present study investigates the effects of supplementing dairy cows with GS from three local red wine varieties. The aim was to evaluate potential changes in milk and cheese composition, with particular attention to chemical, microbiological, sensory, and polyphenolic characteristics, and to assess the feasibility of integrating winery waste into the feed system of local dairy farms. The study highlights an innovative regional circular economy model in which intersectoral synergy between wine and dairy supply chains contributes to a more sustainable, value-added agri-food system.

## 2. Materials and Methods

This preliminary study was conducted under routine management conditions at a privately owned commercial dairy farm in the province of Bolzano, Italy. The dietary supplementation and sample collection did not involve procedures causing pain, suffering, distress, or lasting harm equivalent to or greater than needle insertion. Under D.Lgs. 26/2014, art. 2, this type of activity is outside the scope of the legislation implementing Directive 2010/63/EU. Full informed consent was obtained from the farm owner.

### 2.1. Grape Stalks Pretreatments, Chemical and Elemental Composition Analyses

Grape stalks (GS) were provided by a wine-cellar located in the Trentino-South Tyrol region in Italy. Three red grape varieties, Lagrein (L-GS), Cabernet Sauvignon (CS-GS), and Merlot (M-GS), were collected and stored at −20 °C in our laboratory at Fondazione Edmund Mach (San Michele all’Adige, Italy) until drying. All the GS matrices were dried at 45 °C and size-reduced in a benchtop grinder, except for samples used for chemical analyses. The chemical composition of the treatments was performed at the “LaChi” laboratory, University of Padova (Legnaro, Italy), according to AOAC [18,19] official methods, including dry matter, ash, ether extract, and crude protein (Kjeldahl), as well as fiber fractions (aNDF, ADF, ADL) measured using an Ankom fiber analyzer (Ankom Technology, Rochester, NY, USA). aNDF was determined using heat-stable α-amylase without sodium sulphite. Mineral elements were quantified following AOAC 985.01, 2006.03, and 2011.14 [19] by ICP-OES after microwave-assisted acid digestion (AOAC 2006.03), using external calibration and quality control with blanks and certified reference materials. Treatment composition is shown in Table 1.

### 2.2. Cows, Diets, Feeding, and Management

Four dietary treatments were evaluated in three lactating, multiparous Holstein Friesian cows on a commercial farm (22.1 ± 5.1 days in milk, 3.4 ± 0.50 years of age) using a within-cow experimental design, in which the same animals received all treatments in different experimental weeks. This design did not correspond to a Latin square, and no additional animals were included beyond the three cows described; randomization referred to the allocation of grape stalk supplementation to experimental weeks rather than to animals. The limited number of cows was intentionally chosen, as this study was conceived as a preliminary feasibility trial to evaluate the practical administration and voluntary intake of grape stalks under on-farm conditions. Consequently, statistical power and generalizability were constrained. Cows were housed in individual tie-stalls, fed individually, and milked twice daily using a pipeline milking system. Allocation to treatments was balanced for days in milk and age. The four dietary treatments were as follow: (1) a control diet (CTRL) composed of 22.0 kg DM/day of freshly harvested, single-chop perennial ryegrass pasture offered individually to each cow; (2) a Lagrein grape-stalk diet (L-GS), obtained by supplementing the CTRL with 0.45 kg DM of L-GS; (3) a Cabernet Sauvignon grape-stalk diet (CS-GS), prepared by adding 0.45 kg DM of CS-GS to the CTRL; and (4) a Merlot grape-stalk diet (M-GS), formulated by including 0.40 kg DM of M-GS in the CTRL. Treatments were offered once daily, during a 2-h period following the morning milking at 07:00 A.M. The GS supplementation was provided mixed with the concentrate portion of the diet. The daily concentrate ration (Appendix A Table A1) consisted of 7 kg of concentrate 1 (Optiplus-19; Consorzio Agrario di Bolzano), 2 kg of concentrate 2 (En Mix Maiscobs-Kleie; Consorzio Agrario di Bolzano), and 3 kg of Dry UNIFEED (Dry UNIFEED for dairy cow; Beikircher Grünland Srl, Lana, Italy). The inclusion level of grape stalks was set at approximately 2% of the total dry matter intake (DMI) and was adjusted to maintain a comparable total polyphenols content (TPC) across diets (Appendix A Table A2). As the M-GS showed the highest TPC values, their inclusion rate was slightly reduced to balance the overall polyphenol intake among treatments. This value resulted from the fixed amount of GS provided, mixed with the concentrate portion of the diet, relative to the individually measured total DMI of approximately 22 kg DM per cow per day. Total DMI was calculated as the sum of the dry matter intake from concentrates and hay, both of which were weighed daily and offered individually to each cow. The GS dose was selected based on previous studies reporting potential anti-nutritional effects at higher inclusion rates due to the high polyphenol and lignin content of grape stalks [11], while ensuring palatability, practical feasibility under on-farm conditions, and preventing disruptions to normal feeding behavior. Diets were intentionally formulated to be non-isoenergetic or non-isonitrogenous to reflect realistic on-farm feeding practices and the natural variability in grape stalk composition among wineries. The GS inclusion level was selected based on previous studies reporting potential anti-nutritional effects at higher doses [11], while ensuring palatability and voluntary intake. The GS were dried, chopped, and milled to reduce particle size and improve digestibility and energy availability. The resulting GS powder was stored in large plastic bags from which air was evacuated using a vacuum pump, and the bags were then heat-sealed to ensure airtight conditions. Each bag contained the daily GS portion for a single cow to minimize the risk of aerobic spoilage once opened. The experimental trial lasted seven weeks and followed a structured alternating schedule consisting of control and GS-supplemented weeks. Specifically, cows received the control diet (CTRL) during weeks 1, 3, 5, and 7. Grape stalk supplementation was provided during weeks 2, 4, and 6, with each supplementation week corresponding to a different grape-stalk cultivar: L-GS in week 2, CS-GS in week 4, and M-GS in week 6. The control weeks served as washout periods between supplementation phases. Due to the low inclusion level (2% DM) and the finely ground, homogeneously mixed form of the grape stalk supplement, no specific adaptation period was applied.

### 2.3. Milk Sampling and Quality Traits

Each week, individual milk samples were collected from all cows on the last day of the experimental period and analyzed for microbiological and chemical composition. In addition, 2.5 L of milk per cow was collected for the production of experimental mini-cheeses. In total, 21 milk samples were obtained, and 21 corresponding cheeses were produced.

Milk was collected aseptically according to National Mastitis Council guidelines [20]. Teats were cleaned using single-use wipes; the first streams of foremilk were discarded, and composite milk from all quarters was collected into sterile tubes. One 50 mL aliquot was subdivided and immediately flash-frozen (liquid nitrogen) and stored at −80 °C for microbiological and molecular analyses. A second aliquot was preserved with bronopol and stored at +4 °C until chemical analyses.

Milk samples were analyzed at the Trentingrana Concast laboratory, the main accredited dairy testing facility in the Trento province. The laboratory operates in compliance with ISO/IEC 17025:2017 [21] quality standards. Milk composition parameters, including fat, protein, and lactose, were determined using a Fourier Transform infrared analyzer (FT-IR) following the ISO 9622:2013 [22] accredited method. Casein, urea, and the index of milk aptitude to coagulate (IAC) were also measured using FT-IR, according to validated internal laboratory procedures.

Somatic cell count (SCC) was assessed by flow cytometry following the ISO 13366-2:2006 [23] reference method. All measurements, except for IAC, were calibrated using reference materials produced and supplied by Laboratorio Standard Latte-Associazione Italiana Allevatori (AIA), which operates in conformity with ISO 17034:2016 [24] requirements. The IAC was calculated according to the equation proposed previously [25].

### 2.4. Milk and GS Microbiological Analysis

Milk samples were decimally diluted in sterile peptone water, plated onto selective agar media, and incubated as follows: Plate Count Agar (PCA) supplemented with skim milk (10 g/L, *w*/*v*) for total bacterial aerobic count (TBC), incubated aerobically for 24 h at 30 °C; M17 agar, for cultivating mesophilic and thermophilic cocci-shaped lactic acid bacteria (LAB) count, incubated in anaerobic conditions (using an AnaeroGen^TM^ system), for 48 h at 30 °C and at 45 °C, respectively; de Man, Rogosa and Sharpe (MRS) agar, for mesophilic rod-shaped LAB, maintained in anaerobiosis for 48 h at 30 °C; and Violet Red Bile Agar (VRBA) for coliform enumeration, incubated aerobically at 37 °C for 24 h following the overlay method recommended by the manufacturer.

GS samples were also analyzed microbiologically. Briefly, 1 g of each GS type was homogenized with 9 g of sterile peptone water, serially diluted, plated onto selective agar media, and incubated as follows: PCA with skim milk (10 g/L, *w*/*v*) at 30 °C for 24 h in aerobic conditions, WL (Wallerstein Laboratory) agar with chloramphenicol (0.1 g/L, *w*/*v*) for 6 days at 30 °C, in aerobic conditions, to enumerate yeast and molds and VRBA for coliform enumeration, incubated aerobically at 37 °C for 24 h. All culture media and anaerobic systems were purchased from Oxoid (Thermo Fisher Scientific, Waltham, MA, USA). The data regarding microbiological plate counts were analyzed as means expressed in log CFU/mL.

### 2.5. Milk DNA Extraction, Amplification, and Sequencing

For total genomic DNA extraction, 4 mL of milk was centrifuged at 4000× *g* for 15 min at 4 °C, and the lipid layer was discarded. Genomic DNA was extracted from the pellet using the Qiagen DNeasy Mericon Food Kit (Qiagen, Hilden, Germany), according to the manufacturer’s instructions. The quantification of yield and purity of the extracted DNA was determined by the Nanodrop 8800 fluorospectrometer (Thermo Fisher Scientific).

#### 2.5.1. Preparation of the MiSeq Library

Amplicon library preparation, quality, and quantification of pooled libraries, and pair-end sequencing using the Illumina MiSeq system (Illumina, San Diego, CA, USA) were performed at the Sequencing Platform, Fondazione Edmund Mach (FEM, San Michele all’Adige, Italy). Briefly, for each sample, a 464bp sequence of the V3–V4 region [26,27] of the 16S rRNA gene (*Escherichia coli* positions 341 to 805) was amplified. Unique barcodes were attached before the forward primers to facilitate the pooling and subsequent differentiation of samples. To prevent preferential sequencing of smaller amplicons, the amplicons were cleaned using the Agencourt AMPure kit (Beckman Coulter, Brea, CA, USA) according to the manufacturer’s instructions. Subsequently, the DNA concentrations of the amplicons were determined using the Quant-iT PicoGreen dsDNA kit (Invitrogen, Carlsbad, CA, USA) following the manufacturer’s instructions. In order to ensure the absence of primer dimers and to assay the quality of the purity, the generated amplicon libraries were evaluated by a Bioanalyzer 2100 (Agilent Technologies, Santa Clara, CA, USA) using the High Sensitivity DNA Kit (Agilent Technologies). Following quantification, cleaned amplicons were mixed and combined in equimolar ratios.

#### 2.5.2. Illumina Data Analysis and Sequence Identification by QIIME2

Raw paired-end FASTQ files were demultiplexed using idemp https://github.com/yhwu/idemp/blob/master/idemp.cpp (accessed on 22 July 2025) and imported into Quantitative Insights into Microbial Ecology (Qiime2, version 2020.11). Sequences were quality-filtered, trimmed, de-noised, and merged using DADA2 [28]. Chimeric sequences were identified and removed using the consensus method in DADA2. Representative sequences were aligned with MAFFT and used for phylogenetic reconstruction in FastTree using plugin alignment and phylogeny [29,30]. Taxonomic and compositional analyses were conducted by using plugin feature-classifier https://github.com/qiime2/q2-feature-classifier (accessed on 25 August 2025). A pre-trained Naive Bayes classifier based on the Greengenes 13_8 99% operational taxonomic units (OTUs) database (https://library.qiime2.org/data-resources, accessed on 25 August 2025), which had previously been trimmed to the V4 region of 16S rDNA, bound by the 341F/805R primer pair, was applied to paired-end sequence reads to generate taxonomy tables. The data generated by Illumina sequencing were deposited in the NCBI Sequence Reads Archive (SRA), BioProject accession number PRJNA1311455.

### 2.6. Cheese Making Procedure

Experimental cheeses were produced following a published protocol [31] with minor modifications to obtain mini Caciotta-like cheeses. Milk (2.5 L) was heated to 36 °C and inoculated with starter culture (LYOFASTMOT 086 EE, Sacco, Italy) according to the supplier. Calf rennet (Caglificio Clerici, Cadorago, Italy) was diluted to achieve 51.3 IMCU/L milk (10 mL added per vat). Coagulation occurred within 20–25 min; the curd was cut into nut-sized cubes, cooked at 48 °C for 10 min, rested for 10 min, and whey was drained. The curd was molded and pressed for 30 min with turning every 10 min, then held at 28 °C overnight. Cheeses were brined for 30 min in 20% NaCl, ripened for 1 week at 18 °C, and then for 3 weeks at 5 °C under vacuum.

### 2.7. Cheese Microbiological Analysis

Cheese samples (3 g) were homogenized with 27 g of sterile 2% (*w*/*w*) sodium citrate solution by ULTRA-TURRAX (IKA Werke GmbH and Co. KG, Staufen, Germany) for 5 min at 21,000 rpm inside the microbial cabinet. The homogenates were then serially diluted in sterile peptone water. Samples were plated onto selective agar media and incubated as reported in the milk and GS microbiological analysis chapter.

### 2.8. The Physicochemical Composition and Polyphenol Content of Cheese

Physico-chemical characteristics of cheese samples were analyzed at the end of storage for pH, water activity (ɑ_w_), and total polyphenol content (TPC).

For pH estimation, a 10 g sample of cheese was thoroughly blended with 20 mL of distilled water, and its pH was determined using a pH meter (Hanna Instruments, Woonsocket, RI, USA, model N°HI99191).

After proper calibration, the ɑ_w_ of cheese samples was determined using the 3TE Aqualab water activity meter (Decagon Devices, Pullman, WA, USA).

TPC of GS and cheese were analyzed using the Di Stefano R. and Guidoni S. methods [32], with some modifications as reported in a previous paper [33] and adapted for cheese samples. Briefly, samples were ground and lyophilized before analysis. Polyphenols were extracted from 500 mg of cheese and mixed into 12.5 mL of acidified 70:30 acetone/water solution. The mixture was homogenized, incubated at 40 °C for 10 min, sonicated for 10 min, and shaken thoroughly. Samples were centrifuged at 4000 rpm for 10 min. The supernatant was collected and dried using a rotavapor. Add H_2_SO_4_ 1 N brought to volume in a 25 mL flask. The liquid obtained was then placed in a Falcon tube, centrifuged, and 20 mL was loaded onto the SEP-PAK cartridge. The procedure continues as follows: Wash the cartridge with 2 mL of 0.01 N H_2_SO_4_. Elute into a 20 mL flask with 2 mL methanol and 5 mL water. For the reaction, add 1 mL of FC, wait 3 min, and add 4 mL of 10% Na_2_CO_3_ and make up to the mark with water. Leave to act for 90 min, then read on the spectrophotometer at 700 nm after filtering through a 0.45 µm filter. TPC is reported as mg of catechin per kg of dry matter of cheese.

### 2.9. Color Evaluation

Surface color was quantified in the CIELAB space (L*, a*, b*) using a CR-400 colorimeter (Konica Minolta Sensing Inc., Tokyo, Japan). The instrument was operated with D65 illumination, an observation angle of 2°, and calibrated against a white ceramic reference prior to each session. For each sample, measurements were taken using a Chroma Meter CR-400 colorimeter, supported by CM-S100w SpectraMagic^TM^ colordata software (Konica Minolta Sensing Inc., Tokyo, Japan).

### 2.10. Texture Measurements

Texture was assessed using a TA-XT texture analyzer (Stable Micro Systems, Godalming, Surrey, UK) with a 4 mm cylindrical probe. Samples were tested by compression/penetration at 1.67 mm s^−1^ (trigger force 5 N) to 90% of sample height. Maximum force (Fmax), area under the curve (Auc), and elastic modulus (E; slope of the linear region) were derived from force–deformation curves.

### 2.11. NMR Spectroscopy Analysis of Cheeses

NMR was performed using a modified method [34]. For aqueous extracts, 100 mg lyophilized cheese was mixed with 900 μL deionized water and 100 μL D2O, shaken (20 min, 1200 rpm), centrifuged, filtered (PVDF 0.22 μm), and 600 μL transferred to 5-mm NMR tubes. For lipid extracts, 100 mg of cheese was extracted with 900 μL CDCl_3_, shaken, and filtered as described. Spectra were acquired on a Bruker Avance Neo 400 MHz spectrometer equipped with an autosampler; water suppression was applied to aqueous extracts. Standard automated tuning/shimming and processing were performed in TopSpin. Lipid quantification followed published approaches for linoleic/linolenic acids [35], rumenic acid [36], and triglycerides [37]. Quantification of metabolites was carried out in AssureNMR using an external standard approach (ERETIC/PULCON) [38,39,40,41,42]. Metabolite identification used HMDB and BBIOREFCODE, supported by literature [34,43,44,45,46]

### 2.12. HS-SPME/GC-MS of Cheeses

The technique Head Space—Solid Phase Microextraction/Gas Chromatography—Mass Spectrometry (HS-SPME/GC-MS) was used to analyze volatile organic compounds (VOCs). Freeze-dried samples were suspended in water at 5% (*w*/*v*) concentration and stirred overnight. 3 mL aliquots of the aqueous solutions were transferred into headspace vials (20 mL) capped with PTFE/silicone septa (Agilent Technologies, Santa Clara, CA, USA) and stored at −80 °C until the day of analysis. Three replicates were prepared from each sample. After defrosting, 0.05 mL of an internal standard (aqueous solution of 4-methyl-2-pentanone at 50 mg/L) was added, and the samples were placed into the autosampler of the GC (MPSxt XL, Gerstel, Germany) and stored at 10 °C until analysis. After 10 min at 40 °C, the SPME fiber (2 cm DVB/CAR/PDMS, Supelco, Bellefonte, PA, USA) was exposed to the vial headspace for 40 min. The analytes were then desorbed from the SPME fiber at 240 °C in the injector port of a GC (Agilent Technologies) and separated on a DB-Wax UI fused silica capillary column (30 m × 0.25 mm ID × 0.25 µm film thickness; Agilent Technologies). The carrier gas was Helium, at a constant flow rate of 1.3 mL/min. The GC oven temperature program began at 40 °C for 3 min, then increased to 180 °C at 4 °C per minute, heated for 2 min, then increased to 230 °C at 10 °C per minute, and finally heated at 230 °C for 5 min. The GC was interfaced with a mass spectrometer operating in electron ionization (EI) mode (internal ionization source; 70 eV) with a scan range of *m*/*z* 33–350 (MS 5977A MSD, Agilent Technologies, USA). Compound identification was based on mass spectral matching with the NIST/EPA/NIH (NIST 14) standard and linear retention indices (LRI) compared with the literature. LRI were calculated under the same chromatographic conditions after injection of a C7–C30 n-alkane series (Supelco). Results are reported as semiquantitative concentrations, expressed in µg L^−1^ eq of 4-methyl-2-pentanone.

### 2.13. Statistical Analysis

This study was designed as a preliminary, proof-of-concept investigation. Given the limited number of experimental units (*n* = 3 cows), the large number of response variables, and the practical constraints of a first in-field in vivo study, statistical analyses were conducted at the diet level using aggregated data. Although repeated-measures ANOVA or linear mixed models are generally appropriate for longitudinal designs, their application in this context would require highly parameterized models with unstable variance estimates and limited inferential robustness. Therefore, descriptive statistics and one-way ANOVA (factor = diet) were applied to evaluate differences among treatments. To account for multiple testing across compounds, *p*-values were adjusted using the Benjamini–Hochberg false discovery rate (FDR) procedure. In addition to adjusted significance levels, results are interpreted in terms of effect magnitude and variability to support biological interpretation in this exploratory context. All analyses were performed using R software version 4.1.1 (R Core Team 2021); pairwise differences (letters in the tables) reflect Games–Howell tests within each compound (α = 0.05), which are appropriate under unequal variances and n. Regarding Miseq Illumina data: Alpha-diversity was performed using observed OTU numbers and the Shannon diversity index, and the statistical significance (*p* < 0.05) of alpha-diversity metrics was evaluated using the Kruskal–Wallis H test in QIIME2. Beta-diversity was calculated using unweighted and weighted dissimilarity distance matrices in QIIME2. The beta-diversity distance matrix indicates differences in taxonomic composition between samples, based on either presence/absence or quantitative species abundance data. The output matrix was ordinated using principal coordinate analysis (PCoA) and visualized using EMPeror [47]. Statistical significance of beta-diversity distances between groups was assessed using PERMANOVA (PERmutational Multivariate ANalysis Of VAriance) with 999 permutations in QIIME2. PERMANOVA analyses were performed on both unweighted UniFrac distance matrices, which consider only the presence or absence of taxa, and weighted UniFrac distance matrices, which also account for relative abundance and phylogenetic relationships.

One-way ANOVA was applied using data from CTRL (*n* = 12) and GS groups (*n* = 3 each); when significant differences were detected (*p* < 0.05), pairwise Welch’s *t*-tests with Bonferroni correction were used to account for unequal variances and sample size.

## 3. Results

### 3.1. Grape Stalks Composition

The chemical composition of the three GS samples was comparable across cultivars (Table 1). Fiber fractions represented the main components, with aNDF ranging from 48.75% to 51.73% DM, and ADF from 40.84% to 42.67% DM. Lignin was high in all samples (23.0–26.2% DM). Cellulose and hemicellulose showed a slight increase from L-GS to M-GS. Heavy metal composition of GS is reported in Appendix A, Table A2.

Microbiological counts indicated moderate overall loads. Total aerobic mesophiles (PCA counts) ranged from 4.5 to 5.3 log CFU/mL, with the highest values in CS-GS and the lowest in L-GS. Yeast (WL counts) differed markedly among cultivars and were particularly high in M-GS (5.1 log CFU/mL) compared with L-GS (2.9 log CFU/mL) and CS-GS (2.0 log CFU/mL). Coliform (VRBA counts) were consistently low (2.0–2.5 log CFU/mL) in all samples.

### 3.2. Milk Traits

Milk composition was largely unaffected by GS supplementation (Table 2). Fat, protein, lactose, and casein contents were similar across treatments (*p* > 0.05). The IAC ranged between 103.0 and 109.0, without significant dietary effects (*p* > 0.05).

**Figure A1:**
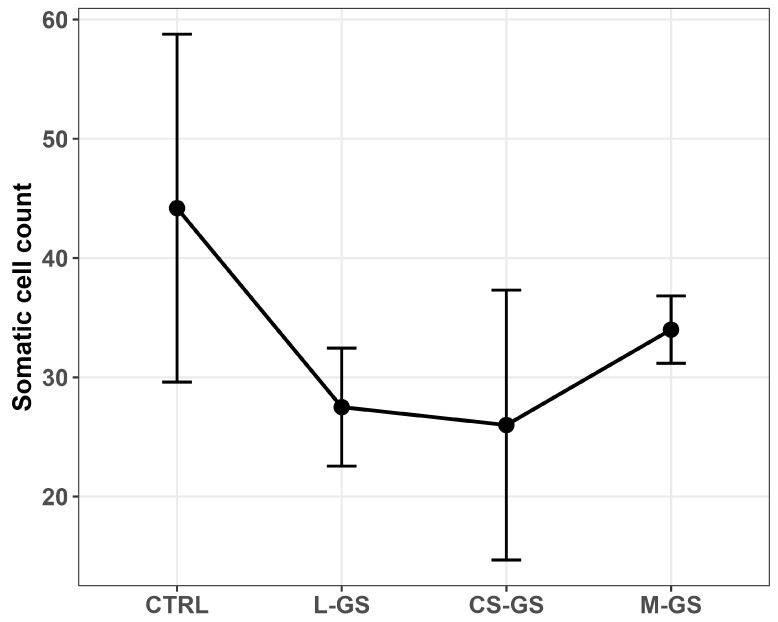
Somatic cell count (SCC) in milk from cows fed the control diet (CTRL) or grape stalk–supplemented diets (L-GS, C-GS, M-GS). Values represent means across experimental weeks within cows.

By contrast, somatic cell count (SCC) and urea showed significant differences. SCC was higher in CTRL (47 × 10^3^ cells/mL) than in L-GS (28 × 10^3^ cells/mL). Somatic cell count (SCC) was significantly higher in CTRL than in GS-supplemented diets (*p* < 0.05; Table 1). For visual support, the SCC trend across diets is shown in Appendix A Figure A1. Urea concentration was highest in CS-GS (27.6 mg/dL) and lowest in M-GS (20.6 mg/dL; *p* < 0.05), although values remained within the physiological range. Milk pH was slightly lower in M-GS than in L-GS and CS-GS (*p* < 0.05), with CTRL showing intermediate values.

### 3.3. Microbial Counts in Milk and Cheeses

Microbial counts from raw milk and cheese are reported in Table 3. In milk, total mesophilic aerobic bacteria (PCA counts) were significantly lower in the M-GS diet (2.8 ± 0.46 log CFU/mL) compared to CTRL (4.5 ± 0.71) (*p* < 0.05). The average PCA across all GS diets (GS TOT) was 4.0 ± 1.09 log CFU/mL. Mesophilic lactobacilli (MRS-30 counts) were significantly lower in M-GS (3.2 ± 0.13) and L-GS (3.3 ± 0.16) than in CS-GS (3.9 ± 0.30; *p* < 0.05). Mesophilic lactococci (M17-30 counts) also showed significant diet-related differences: CTRL and CS-GS showed higher counts (5.0 ± 1.33 and 4.8 ± 0.47, respectively) than L-GS (3.6 ± 0.07) and M-GS (4.0 ± 0.28). Thermophilic lactococci (M17-45 counts) and coliforms (VRBA counts) did not differ significantly among diets and remained low in milk.

In cheese samples, microbial counts increased as expected following manufacture and ripening. PCA counts were highest in L-GS cheeses (9.7 ± 0.27), significantly higher than M-GS (8.5 ± 0.08) and slightly higher than CTRL and CS-GS cheeses (9.1 ± 0.46 and 9.4 ± 0.13, respectively). No significant diet effect was observed for cheese LAB counts (MRS-30, M17-30, M17-45) and coliforms. Coliforms remained under 5 log CFU/g in all treatments (3.9–4.6 log CFU/g).

### 3.4. Milk Microbiota Composition by MiSeq Illumina

**Figure 1:**
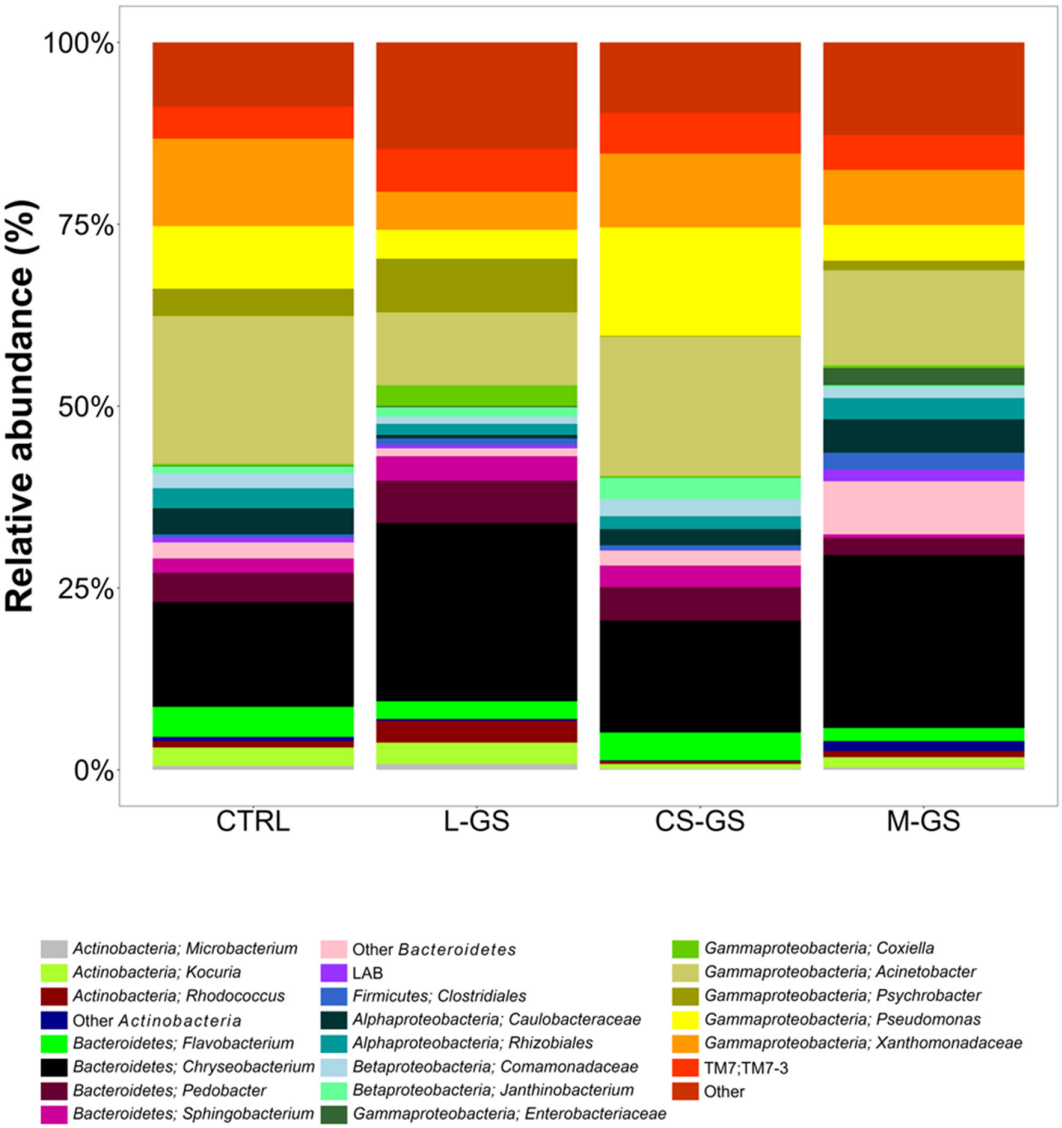
Taxa Composition expressed as relative abundance of raw milk microbiota in dairy cows fed with four diets: CTRL, control diet; L-GS, Lagrein Grape Stalk supplement; CS-GS, Cabernet Sauvignon Grape Stalk supplement; M-GS, Merlot Grape Stalk supplement.

High-throughput sequencing of the 16S rRNA gene (Figure 1) revealed a diverse bacterial community in raw milk samples from cows fed CTRL or GS-supplemented diets (L-GS, CS-GS, M-GS). Detected taxa belonged mainly to Proteobacteria, Bacteroidetes, Actinobacteria, Firmicutes, and the candidate phylum TM7. Only taxa with relative abundance above 0.1% in at least one dietary group are reported in Figure 1.

Across all samples, Gammaproteobacteria dominated, particularly *Acinetobacter*, which accounted for 34.0% in CTRL, 31.7% in CS-GS, 21.5% in M-GS, and 18.0% in L-GS.

*Chryseobacterium* (Bacteroidetes) was the second major genus, with relative abundances of 18.7% (CTRL), 25.1% (L-GS), 17.9% (CS-GS), and 34.3% (M-GS). *Psychrobacter* was most abundant in CTRL (4.8%) and very low in GS-supplemented groups (0.16% in L-GS, undetectable in CS-GS, and 1.9% in M-GS). *Pseudomonas* showed the highest relative abundance in CS-GS (18.0%), intermediate abundances in CTRL (6.5%), and much lower abundances in L-GS (1.0%) and M-GS (0.8%).

LAB remained at low relative abundances in all samples but was higher in M-GS (2.1%) than in CTRL (0.6%), L-GS (0.5%), and CS-GS (0.1%). *Clostridiales* increased in GS-fed groups, with 3.5% in M-GS, 1.7% in L-GS, compared to 0.6% in CTRL and 0.1% in CS-GS. Several genera likely related to environmental exposure or feed-associated sources were also detected: *Kocuria* and *Rhodococcus* (Actinobacteria) were more abundant in CTRL (4.2% and 1.2%, respectively) and L-GS (2.6% and 3.8%) than in the other groups. *Flavobacterium* was relatively stable (4.2–4.9%) in CTRL, L-GS, and CS-GS, but decreased s in M-GS (0.6%). *Pedobacter* was absent in M-GS but reached 5.3% in L-GS. Low-abundance taxa (1–3%) included *Janthinobacterium*, *Comamonadaceae*, *Caulobacteraceae*, and *Xanthomonadaceae*. The candidate phylum TM7 (c__TM7-3, now classified within Saccharibacteria), was detected in all treatments with relative abundances of 4.9% (CTRL), 12.5% (L-GS), 3.7% (CS-GS) and 6.0% (M-GS).

Cheeses in this study were produced using defined commercial starter cultures. Illumina sequencing was not applied to cheese; thus, microbial carryover was only inferred indirectly.

#### Milk Microbiota Alpha- and Beta-Diversity

Alpha-diversity metrics are reported in Table 4. OTU richness differed significantly among diets (*p* = 0.012), with the highest number of observed taxa in CS-GS (451 ± 41) and the lowest in M-GS (161 ± 20). Shannon diversity, which considers both richness and evenness, also differed significantly among groups (*p* = 0.0015) and was the highest in CS-GS (0.787 ± 0.003). Evenness did not differ significantly (*p* = 0.621), indicating that although the number of OTUs varied, the relative distribution of abundance within communities was similar across diets.

PERMANOVA based on unweighted UniFrac distances did not reveal significant differences among dietary groups, suggesting that most taxa were shared across treatments. In contrast, PERMANOVA analysis based on weighted UniFrac distances (Table 5), showed a significant difference between CTRL and M-GS (Pseudo-F = 3.381, *p* = 0.023). No other pairwise comparisons were significant at *p* < 0.05, in agreement with previous observations from alpha diversity and taxonomic profiles. These patterns are consistent with the observed shifts in relative abundance, particularly in M-GS, where LAB and *Clostridiales* increased, and spoilage-associated genera such as *Psychrobacter* and *Pseudomonas* decreased.

### 3.5. Physicochemical Composition and Polyphenol Content of Cheese

GS-supplementations in the diet of dairy cows induced minor but measurable changes in cheese’s physicochemical properties, with some cultivar-specific effects (Table 6). Cheeses’ pH ranged from 5.17 to 5.24, with no significant differences among diets.

Water activity (ɑ_w_) differed significantly; M-GS cheeses showed significantly lower ɑ_w_ (0.940 ± 0.015) than L-GS cheeses (0.974 ± 0.005, *p* < 0.05), with CTRL and CS-GS cheeses showing intermediate values.

TPC was highest in CS-GS cheeses (224 ± 34 mg/kg), followed by CTRL (211 ± 34 mg/kg), but these differences were not statistically significant.

Cheese color parameters were only slightly affected. Lightness (L*) did not differ among treatments. The a* coordinate (green–red axis) was significantly more negative in M-GS cheese (−2.69 ± 0.30) than in CTRL (−1.63 ± 0.51), indicating a shift toward a higher green component. The b* coordinate (yellow–blue axis) was slightly lower in CS-GS cheese (10.36 ± 1.8) than in L-GS (12.38 ± 1.8; *p* < 0.05), suggesting a less intense yellow color.

### 3.6. NMR Metabolites; Aqueous and Lipid Fractions in Cheeses

Targeted ^1^H-NMR of cheese aqueous and lipid fractions quantified organic acids, amino acids, sugars, choline species, and selected minor metabolites (Table 7).

Among carbohydrates, glucose was significantly lower in L-GS than in CTRL cheeses. Lactic acid concentrations did not differ significantly among diets, whereas acetic acid was higher in L-GS (69.0 ± 9.5 mg/100 g) and lower in M-GS (47.8 ± 10.6 mg/100 g). Among minor alcohols, ethanol was higher in L-GS than in M-GS, reinforcing the acetate trend and supporting a more heterofermentative profile under L-GS, while glycerol was lower in L-GS than in CTRL. Most free amino acids (glycine, methionine, GABA, proline, tyrosine, serine, valine) did not significantly differ among diets. GABA showed only slightly higher mean values in L-GS. Choline was significantly lower in M-GS than in all the other cheeses. 2,3-butanediol did not significantly differ among diets.

In the lipid fraction (Table 7), differences in the major classes were small, with UFA and MUFA varying by less than 0.8%, and CLA ranged from 2.7 to 3.0%. M-GS cheeses showed a trend toward slightly higher MUFA and CLA.

### 3.7. Volatile Organic Compounds in Cheese

Volatile organic compounds (VOCs) in cheeses from the CTRL, L-GS, CS-GS, and M-GS diets are reported in Table 8.

Among SCFAs, butanoic acid showed a strong diet effect: all GS treatments had significantly lower concentrations than CTRL (1.878–2.587 mg/kg vs. 5.440 mg/kg).

Heptanoic acid exhibited a bidirectional pattern with concentrations approximately 66% lower in L-GS and 52% higher in M-GS than in CTRL. Several pairwise contrasts were significant, indicating cultivar-specific effects on lipolytic and β-oxidation pathways during ripening. Hexanoic and octanoic acids tended to be lower in both L-GS and M-GS than in CTRL cheeses, although some of these differences did not reach significance after multiple-comparison correction.

Among aldehydes, hexanal, a marker of lipid oxidation, varies by diet. CS-GS showed a strong and significant reduction (−75% vs. CTRL), whereas M-GS values were slightly higher and L-GS slightly lower than CTRL. The Strecker aldehydes 2-methylbutanal and 3-methylbutanal remained within a narrow concentration range and were not significantly affected by diet.

Within ketones, 2-pentanone showed a significant overall diet effect with a tendency to increase in L-GS (+77% vs. CTRL), whereas 2-heptanone and 2-nonanone did not differ significantly. Acetoin concentrations were significantly lower in CS-GS and L-GS than in CTRL cheeses.

Regarding esters, CTRL cheeses had significantly higher concentrations of ethyl hexanoate, ethyl octanoate, and ethyl decanoate than all GS treatments. Given the low odor thresholds of several ethyl esters in hydroalcoholic matrices (e.g., ethyl hexanoate ~0.014 mg/L; ethyl octanoate ~0.005 mg/L), even modest reductions may be sensorially relevant.

Ethanol values should be interpreted with caution, as ethanol was used during cheese cutting for surface sterilization and may have contributed to contamination.

## 4. Discussion

The chemical composition of the three GS types confirmed a highly fibrous, lignified matrix with limited protein and lipid contribution, in agreement with previous characterizations of grape-derived residue [48]. Ash content varied slightly among cultivars, likely reflecting differences in vineyard soil composition and mineral accumulation during vinification. Elevated lignin content, high aNDF and ADF values indicate low intrinsic rumen degradability, but the presence of polyphenols supports their potential use as functional feed ingredients within circular economy strategies [9,49]. Moderate microbial loads and low coliform counts indicate good hygienic quality of the collected GS, making them suitable for controlled dietary inclusion. It should be noted, however, that these observations are based on a limited experimental scale and should therefore be interpreted as indicative rather than definitive.

The absence of major changes in milk fat, protein, lactose, and casein confirms that GS supplementation at the inclusion levels used was metabolically well tolerated and did not impair the energy–protein balance needed for milk synthesis. These outcomes are consistent with studies reporting that inclusion of grape-residue silage in dairy cow diets did not affect milk yield or the concentrations of protein, fat, and lactose [50]. Nevertheless, given the small number of animals and the short feeding periods, subtle diet-related effects on milk synthesis cannot be excluded and may require longer-term trials to be fully resolved.

The reduction in SCC observed in GS-fed cows may reflect a potential benefit of GS on udder health, possibly linked to the antioxidant and anti-inflammatory properties of grape polyphenols [51]. Feeding strategies that increase antioxidant intake have been associated with lower SCC and improved mammary health status [14]. Differences in milk urea among treatments may reflect subtle shifts in rumen nitrogen dynamics and protein degradability, as polyphenol-rich feeds can modulate rumen proteolysis and nitrogen recycling [52]. However, all values remained within physiological limits, indicating only mild adjustments in protein metabolism. The reduction in total mesophilic aerobic counts in M-GS milk suggests that GS, particularly from the Merlot cultivar, may exert mild antimicrobial effects at the farm level.

In cheese samples, microbial counts increased as expected during ripening. The reduced total aerobic bacteria counts in M-GS cheeses support the hypothesis that GS, and, in particular, M-GS, may contribute to antimicrobial activity, potentially through polyphenols and tannins [10,13]. Mesophilic LAB in milk also showed some sensitivity to GS supplementation, particularly in L-GS and M-GS groups, but this effect was not retained in the cheese, where LAB counts remained high and stable. This suggests that any inhibitory action of dietary polyphenols on LAB during milking is overcome by the cheesemaking process, starter addition, or ripening conditions [53]. Coliform counts remained stable and low in both milk and cheese, indicating no hygiene or safety issues associated with GS inclusion. Overall, the microbial results suggest that GS can be safely integrated into dairy cows’ diets without detrimental effects on milk hygiene or cheese microbial stability, while potentially offering antimicrobial benefits in raw milk. These findings support the role of GS as a functional feed ingredient within a circular bioeconomy model, linking viticulture and dairy sectors [54].

MiSeq data showed that GS supplementation modulated the relative abundances of dominant genera rather than introducing new taxa, consistent with the concept of a conserved core milk microbiota [55]. *Acinetobacter* is a well-documented psychrotrophic genus associated with cold storage spoilage and biofilm formation in dairy environments [56]. Its high abundance across treatments reflects its ubiquity in raw milk systems and also suggests partial suppression under GS supplementation, particularly in L-GS. *Chryseobacterium* (Bacteroidetes), frequently isolated from milking equipment and moist environments and previously described in raw milk collected under farm conditions [57], was prevalent in all samples. Its higher levels in GS-fed cows may reflect transfer from the stalks or changes in teat/skin microbiota.

*Psychrobacter*, another spoilage-associated genus, was most abundant in CTRL, supporting the idea that GS supplementation, especially CS-GS and L-GS, may suppress *Psychrobacter* populations. This aligns with reports where grape polyphenols exert antimicrobial effects on Gram-negative psychrotrophs [58]. Similarly, *Pseudomonas* was highest in CS-GS, elevated in CTRL, but reduced in L-GS and M-GS, suggesting cultivar-specific effects. The high abundance in CS-GS may reflect post-milking contamination, whereas its suppression under Lagrein and Merlot stalk supplementation may indicate polyphenol-driven inhibition.

LAB had a low relative abundance but increased modestly in the M-GS compared with the other groups. Although the values were small, such a shift could influence cheese fermentation and microbial balance, as LAB contribute to milk preservation and can outcompete undesirable taxa [59]. The increase under M-GS supplementation may be due to changes in rumen fermentation and milk composition that favor LAB survival. *Clostridiales*, including spore-forming bacteria, showed higher levels in the GS-fed groups than in CTRL. This may be linked to the fibrous nature of grape stalks or to the presence of spores naturally associated with stalk biomass [60]. Although levels remained within safe limits, the rise in spore-formers warrants attention due to potential risks to cheese quality, such as late-blowing defects [61].

Several taxa likely reflected environmental or feed-derived microbes. *Kocuria* and *Rhodococcus* (Actinobacteria) were more represented in CTRL and L-GS, but less in the other groups. These genera are typically associated with skin and soil, suggesting cow- or bedding-related origin [62]. *Flavobacterium* remained relatively stable in CTRL, L-GS, and CS-GS but decreased in M-GS, suggesting a possible exclusion mechanism or a feed-dependent ecological shift. *Pedobacter* was not detected in M-GS but was more abundant in L-GS, suggesting a cultivar-linked pattern. As a typical soil and rhizosphere genus, *Pedobacter* may reflect microbial carryover from grape stalks, which can host plant- and vineyard-associated microbiota [63]. Low-abundance taxa such as *Janthinobacterium*, *Comamonadaceae*, *Caulobacteraceae*, and *Xanthomonadaceae*, which are commonly associated with moist surfaces or plant environments and are frequently reported in raw milk, contribute to overall diversity and may act as early colonizers or biofilm-forming members [64,65]. The presence of TM7 (c__TM7-3) although still poorly understood, is typically associated with low-biomass environments and may influence microbial interactions or community stability [66].

Overall, the milk microbiota of cows fed with GS-supplemented diets showed clear compositional shifts, with both consistent and cultivar-dependent patterns. L-GS milk showed lower levels of *Acinetobacter* and *Psychrobacter* alongside an increase in *Pedobacter* and *Rhodococcus*; CS-GS milk was characterized by higher *Pseudomonas* and a moderate suppression of other taxa; M-GS milk displayed the highest representation of LAB together with an increase in *Clostridiales*, suggesting a combination of beneficial and cautionary microbial effects. These findings highlight how GS inclusion can influence the milk microbiome, supporting its use in sustainable dairy systems. Modulating spoilage taxa while modestly promoting fermentative microbes aligns with circular economy goals and offers a functional advantage in raw milk quality. These taxonomic shifts are consistent with literature showing that polyphenol-rich by-products, such as grape pomace or stalks, can modulate the ruminal microbiota and alter downstream microbial ecology, including in milk [11,14]. Given the limited sample size and temporal scope of the study, these taxonomic shifts should be interpreted as trends rather than conclusive evidence of diet-driven microbiome restructuring.

Differences in alpha diversity further support a cultivar-dependent effect. OTU richness was highest in CS-GS and lowest in M-GS, suggesting either a richness-promoting effect or a more selective microbial response depending on the cultivar. Shannon diversity confirmed these trends, whereas evenness did not vary significantly, indicating that the distribution of abundances among taxa was similar across treatments. These results agree with previous reports that grape-derived polyphenols can either support microbial diversity at low concentrations or exert selective pressure at higher doses [10,11]. The discrepancy between unweighted and weighted UniFrac results underscores that GS supplementation reshaped the abundance patterns of existing taxa, rather than introducing distinct microbial lineages. This is ecologically possible, as the milk microbiota is largely conserved at the core level, with diet, environment, and farm management, rather than the community composition, primarily influencing the relative abundances of taxa [55].

The overall stability of cheese pH across treatments indicates that GS supplementation did not disrupt acidification or lactic fermentation. The lower ɑ_w_ in M-GS cheeses may reflect slight differences in moisture retention due to feed-induced changes in milk composition or curd formation. Similar effects have been reported with olive cake supplementation, where changes in milk protein content and fat composition influenced cheese moisture and water activity [67]. The higher TPC in CS-GS cheeses suggests some carryover of dietary polyphenols into the cheese matrix. Transfer of polyphenols from diet to cheese has been reported in other studies, using grape pomace or olive phenolic-enriched feeds, with elevated TPC and antioxidant capacity retained after ripening [68]. Small shifts in color coordinates (more negative a* in M-GS; slightly lower b* in CS-GS) may reflect pigment-protein–polyphenol interactions during ripening, as described for other plant polyphenol sources [69]. These effects were small in magnitude and should be regarded as supportive rather than demonstrative evidence of dietary transfer.

Targeted ^1^H-NMR profiling of cheeses revealed modest but coherent metabolite differences, mainly linked to carbohydrate-fermentative metabolism and membrane-related compounds with clear, cultivar-dependent trends. Glucose was significantly lower in L-GS than in CTRL, suggesting enhanced glucose utilization by the cheese microbial under L-GS. During ripening, residual lactose is hydrolysed to glucose and galactose and typically converted to lactate by homofermentative LAB; however, citrate-positive and heterofermentative pathways can redirect carbon toward acetate, ethanol, and CO_2_ (along with diacetyl/acetoin) depending on redox status and microbial composition [70,71]. The overall stability of most free amino acids indicates robust proteolysis across treatments, reflecting the resilience of milk enzymes, residual coagulant, and LAB peptidases to moderate diet changes [70]. The selective decrease in choline in M-GS cheeses suggests altered phospholipid turnover or microbial utilization of choline-type osmolytes, in agreement with reports where choline species are sensitive markers of membrane-related processes in dairy matrices [72]. Small lipidomic shifts (slightly higher MUFA and CLA in M-GS) are consistent with the potential of grape by-products to affect rumen biohydrogenation and lipid oxidative stability [73,74], although the limited magnitude and lack of replicated variance call for cautious interpretation. This profile may be indirectly influenced by differences in milk microbiota (e.g., different LAB presence and lower spoilage bacteria), although it is more likely driven by starter strains’ activity modulated by substrate availability (e.g., residual lactose, citrate) and redox balance. Similar, subtle fermentation shifts have been reported in cheeses from cows fed agro-industrial by-products, which can alter ruminal fermentation and milk composition [73,75]. More generally, cheese NMR metabolomics identifies organic acids (lactate, acetate, citrate), residual sugars (glucose and galactose), choline species, and selected amino acids as dominant aqueous features that reflect ripening stage, microflora, and processing conditions [76].

Diet also influenced cheese VOC profiles, particularly SCFAs, aldehydes, methyl ketones, and esters. Among SCFAs, major contributors to pungent, sweaty, or animal-like notes [77], GS supplementation markedly reduced butanoic acid, with parallel downward trends in hexanoic and octanoic acids in L-GS and M-GS cheeses. This attenuation of pungent SCFA notes is consistent with the lower abundance of psychrotrophic, lipolytic bacteria such as *Psychrobacter* and *Pseudomonas* in the corresponding milk [78,79]. Polyphenol-rich grape by-products can influence rumen biohydrogenation, microbial ecology, and, downstream, the availability of substrate for lipolysis and β-oxidation in cheese. Several studies and reviews report improved oxidative stability and altered lipid composition in milk or dairy products after grape by-product supplementation, providing a plausible upstream explanation for the observed SCFA changes [80,81].

Aldehydes also responded to dietary treatment. Hexanal, a marker of lipid oxidation, was strongly suppressed in CS-GS cheeses, consistent with improved oxidative stability associated with grape-derived polyphenols [82,83]. The Strecker aldehydes 2-methylbutanal and 3-methylbutanal, derived from microbial catabolism of isoleucine and leucine, respectively, remained within a narrow concentration range and were not significantly affected by diet, suggesting that diet-induced effects on amino acid-derived aroma pathways were minor compared with those from lipid-derived routes. This agrees with the dominant role of lipolysis and oxidative processes in shaping the aroma of young and semi-ripened cheeses [84]. Methyl ketones arise via β-oxidation–decarboxylation of fatty acids, classically abundant in blue cheeses but also present across styles [85]. Acetoin was lower in GS cheeses (particularly CS-GS), moderating buttery notes, and L-GS cheeses tended to show higher 2-pentanone, but overall shifts were modest. Overall, the effects of GS supplementation on cheese metabolites were small but showed consistent cultivar-specific patterns that align with known pathways of cheese ripening. These trends suggest that GS influences fermentation dynamics in predictable ways. Future work with a larger sample size, full replicate lipid analyses, and deeper functional microbial profiling (e.g., metagenomics or metatranscriptomics) would allow a clearer identification of the microbial or enzymatic mechanisms responsible for these metabolite shifts.

In conclusion, GS supplementation generally softened pungent and rancid aroma notes (strong butanoic-acid decrease; downward trends in hexanoic and octanoic acids in L-GS and M-GS), while CS-GS uniquely suppressed hexanal, suggesting a less “green” profile (less oxidized). Small reductions in ethyl esters in GS cheeses may also have contributed to a slightly less fruity profile. Although changes in individual VOCs were moderate, the consistent directional trends across SCFAs, aldehydes, methyl ketones, and esters support the view that GS supplementation can modulate cheese volatile profiles through plausible mechanisms involving lipolysis, oxidation, and microbial metabolism. Future work should verify the sensory relevance of these chemical shifts and further relate them to milk composition and microbial ecology.

## 5. Conclusions

This study provides an integrated evaluation of how dietary inclusion of Grape Stalks from three different red cultivars (Lagrein, Cabernet Sauvignon, and Merlot) can affect milk composition and cheese biochemistry within a regional circular economy framework. While GS supplementation produced only modest effects on bulk milk traits, it consistently modulated downstream indicators of fermentation, lipid oxidation, and microbial metabolism in cheese.

Cheeses from GS-supplemented cows displayed cultivar-dependent metabolic signatures. Lagrein GS promoted a shift toward a slightly more heterofermentative ripening profile, reflected by higher acetate and ethanol and lower glucose and glycerol. In contrast, Merlot GS was associated with reduced choline levels and lower acetate and ethanol levels, suggesting differences in membrane-associated metabolism. VOCs analyses confirmed these biochemical patterns: all GS diets markedly reduced butanoic acid, softening rancid and pungent notes, while Cabernet Sauvignon GS uniquely suppressed hexanal, suggesting enhanced oxidative stability.

Overall, these results suggest that GS supplementation can subtly reshape the biochemical and aromatic profiles of cheese without compromising milk quality or technological performance. Importantly, the present work should be regarded as a preliminary, exploratory investigation designed to assess the feasibility and potential of grape-stalk inclusion in dairy cow diets. As such, the observed trends warrant confirmation through more comprehensive experimental designs. Future research should therefore include trials involving a larger number of animals, longer supplementation periods, and replicated farm conditions to strengthen statistical robustness and biological interpretation. In addition, the use of mixed grape-stalk fractions, more closely reflecting the heterogeneous winery residues typically generated during winemaking, would improve the practical relevance of this feeding strategy. Sensory evaluation will be essential to determine the perceptual significance of the observed chemical shifts, while multi-omics approaches could further elucidate how feed-derived polyphenols interact with microbial communities and metabolic pathways during cheesemaking.

These findings highlight the potential of winery by-products as functional feed ingredients, able to link viticulture and dairy production in a sustainable, value-added circular system. This strategy is particularly well-suited to regions such as Trentino Alto Adige, where vineyards and dairy farms operate in proximity, enabling efficient local reuse of agricultural residues.

## Figures and Tables

**Table 1:** Chemical and microbiological composition of grape stalks. L-GS, Lagrein Grape Stalk; CS-GS, Cabernet Sauvignon Grape Stalk; M-GS, Merlot Grape Stalk.

	L-GS	CS-GS	M-GS
Chemical Composition			
DM (g/100 g)	94.07 ± 0.02	94.71 ± 0.07	95.29 ± 0.14
Ash (% DM)	7.71 ± 0.06	11.09 ± 0.08	9.88 ± 0.06
Protein (% DM)	7.91 ± 0.08	5.13 ± 0.14	4.10 ± 0.01
EE (% DM)	3.01 ± 0.21	1.93 ± 0.26	2.13 ± 0.22
FG (% DM)	18.97 ± 0.08	19.31 ± 0.73	22.88 ± 1.51
aNDF (% DM)	48.75 ± 0.17	49.28 ± 0.76	51.73 ± 0.65
ADF (% DM)	40.96 ± 0.67	40.84 ± 0.88	42.67 ± 0.40
AIA (% DM)	0.31 ± 0.26	0.45 ± 0.13	0.43 ± 0.08
ADL (% DM)	26.22 ± 0.99	23.04 ± 0.47	23.65 ± 0.72
Hemicellulose (% DM)	7.79 ± 0.50	8.44 ± 0.12	9.06 ± 0.25
Cellulose (% DM)	14.42 ± 0.06	17.35 ± 0.29	18.59 ± 0.40
Lignin (% DM)	26.22 ± 0.99	23.04 ± 0.47	23.65 ± 0.72
Microbiological Composition			
PCA (log CFU/g)	4.5	5.3	5.0
WL (log CFU/g)	2.9	2.0	5.1
VRBA (log CFU/g)	2.5	2.0	2.1

Abbreviations: DM, dry matter; EE, ether extract; FG, crude fiber; aNDF, neutral detergent fiber; ADF, acid detergent fiber; AIA, Acid Insoluble Ash; ADL, Acid Detergent Lignin; PCA, plate count agar; WL, Wallerstein Laboratory; VRBA, Violet Red Bile Agar.

**Table 2:** Effect of diet system on chemical composition in milk samples collected from individual cows clustered in four diets: CTRL, control diet; L-GS, Lagrein Grape Stalk supplement; CS-GS, Cabernet Sauvignon Grape Stalk supplement; M-GS, Merlot Grape Stalk supplement.

	CTRL	L-GS	CS-GS	M-GS
Fat (g/100 g)	3.4 ± 0.71	3.3 ± 1.42	2.7 ± 0.11	3.1 ± 0.43
Protein (g/100 g)	3.4 ± 0.25	3.4 ± 0.25	3.5 ± 0.25	3.4 ± 0.19
Lactose (g/100 g)	4.8 ± 0.18	4.8 ± 0.13	4.9 ± 0.15	4.7 ± 0.30
Casein (g/100 g)	2.6 ± 0.090	2.5 ± 0.028	2.6 ± 0.064	2.6 ± 0.17
SCC (×10^3^ cell/mL)	47 ± 20 ^B^	28 ± 5 ^A^	26 ± 11 ^AB^	34 ± 3 ^AB^
Urea (mg/dL)	24.0 ± 4.0 ^AB^	22.8 ± 2.1 ^AB^	27.6 ± 3.0 ^B^	20.6 ± 3.0 ^A^
IAC	103.0 ± 6.1	105.3 ± 5.5	109.0 ± 7.8	105.3 ± 5.7
pH	6.51 ± 0.06 ^AB^	6.64 ± 0.03 ^B^	6.57 ± 0.04 ^B^	6.43 ± 0.08 ^A^

For each column, values with different superscript letters are significantly different (*p* < 0.05, one-way Anova with post hoc Tukey HSD). Abbreviations: IAC = Index of milk aptitude to coagulate; SCC = Somatic Cell Count.

**Table 3:** Microbial counts of raw milk (expressed as log CFU/mL) and related cheese samples (expressed as log CFU/g) collected from individual cows clustered in four diets: CTRL, control diet; L-GS, Lagrein Grape Stalk supplement; CS-GS, Cabernet Sauvignon Grape Stalk supplement; M-GS, Merlot Grape Stalk supplement.

	CTRL	L-GS	CS-GS	M-GS
Milk				
PCA	4.5 ± 0.71 ^B^	4.9 ± 0.15 ^B^	4.3 ± 0.95 ^B^	2.8 ± 0.46 ^A^
MRS-30	3.8 ± 0.52 ^AB^	3.3 ± 0.16 ^A^	3.9 ± 0.30 ^B^	3.2 ± 0.13 ^A^
M17-30	5.0 ± 1.33 ^B^	3.6 ± 0.065 ^A^	4.8 ± 0.47 ^AB^	4.0 ± 0.28 ^A^
M17-45	3.2 ± 0.46 ^A^	3.3 ± 0.14 ^A^	2.9 ± 0.24 ^A^	2.8 ± 0.33 ^A^
VRBA	2.3 ± 0.85 ^A^	2.3 ± 0.039 ^A^	2.5 ± 0.30 ^A^	2.4 ± 0.37 ^A^
Cheese				
PCA	9.1 ± 0.46 ^AB^	9.7 ± 0.27 ^B^	9.4 ± 0.13 ^AB^	8.5 ± 0.080 ^A^
MRS-30	8.8 ± 0.43 ^A^	9.1 ± 0.34 ^A^	9.3 ± 0.19 ^A^	8.6 ± 0.46 ^A^
M17-30	9.0 ± 0.46 ^A^	9.0 ± 0.29 ^A^	9.4 ± 0.17 ^A^	8.7 ± 0.38 ^A^
M17-45	8.7 ± 0.43 ^A^	8.9 ± 0.34 ^A^	8.6 ± 0.51 ^A^	8.2 ± 0.26 ^A^
VRBA	4.4 ± 0.65 ^A^	3.9 ± 0.51 ^A^	4.4 ± 0.15 ^A^	4.6 ± 0.35 ^A^

For each column, bacterial count values with different superscripts are significantly different (*p* < 0.05, one-way Anova with post hoc Tukey HSD). Abbreviations: PCA: total mesophilic aerobic bacteria; MRS-30: total mesophilic lactobacilli; M17-45: total thermophilic lactococci; M17-30: total mesophilic lactococci; VRBA: total coliforms.

**Table 4:** Alpha diversity metrics (mean ± SD) and ANOVA (F and *p* values) for milk microbiota across dietary treatments: CTRL, control diet; L-GS, Lagrein Grape Stalk supplement; CS-GS, Cabernet Sauvignon Grape Stalk supplement; M-GS, Merlot Grape Stalk supplement.

Metric	CTRL	L-GS	CS-GS	M-GS	F Value	*p* Value
OTUs	305 ± 85 ^B^	331 ± 72 ^B^	451 ± 41 ^C^	161 ± 20 ^A^	7.21	0.012
Evenness	0.758 ± 0.327 ^A^	0.692 ± 0.402 ^A^	0.893 ± 0.010 ^A^	0.815 ± 0.110 ^A^	0.62	0.621
Shannon	0.666 ± 0.218 ^A^	0.747 ± 0.017 ^B^	0.787 ± 0.003 ^C^	0.720 ± 0.077 ^B^	14.08	0.0015

For each column, bacterial count values with different superscripts are significantly different (*p* < 0.05, one-way Anova with post hoc Tukey HSD).

**Table 5:** PERMANOVA analysis (999 permutations) results based on weighted UniFrac distances, for bacterial communities in raw milk samples from dairy cows fed with four diets: CTRL, control diet; L-GS, Lagrein Grape Stalk supplement; CS-GS, Cabernet Sauvignon Grape Stalk supplement; M-GS, Merlot Grape Stalk supplement.

Pairwise Comparisons for Diet	Weighted UniFrac	
	Pseudo-F	*p*-Value
CTRL vs. L-GS	1.522	0.214
CTRL vs. CS-GS	0.518	0.810
CTRL vs. M-GS	3.381	0.023 *
L-GS vs. CS-GS	5.386	0.263
L-GS vs. M-GS	1.343	0.336
CS-GS vs. M-GS	6.560	0.096

Significance levels: * *p* < 0.05.

**Table 6:** Mean values (*n* = 3) for the pH, ɑ_w_, Total polyphenol content (TPC, mg/Kg), primary and secondary texture parameters and color evaluation parameters of the ripened cheeses obtained by raw milk from dairy cows fed with four diets: CTRL, control diet; L-GS, Lagrein Grape Stalk supplement; CS-GS, Cabernet Sauvignon Grape Stalk supplement; M-GS, Merlot Grape Stalk supplement.

	CTRL	L-GS	CS-GS	M-GS
pH	5.24 ± 0.09	5.22 ± 0.04	5.17 ± 0.04	5.20 ± 0.12
ɑ_w_	0.963 ± 0.014 ^AB^	0.974 ± 0.005 ^B^	0.968 ± 0.005 ^AB^	0.940 ± 0.015 ^A^
TPC	211 ± 34	190 ± 23	224 ± 34	194 ± 34
Color parameters				
L	74.12 ± 4.0	73.83 ± 4.7	73.01 ± 5.2	73.04 ± 5.6
a	−1.63 ± 0.51 ^A^	−1.71 ± 0.73 ^A^	−1.95 ± 0.33 ^A^	−2.69 ± 0.30 ^B^
b	12.05 ± 2.2 ^AB^	12.38 ± 1.8 ^B^	10.36 ± 1.8 ^A^	12.06 ± 3.5 ^AB^

For each row, values with A and B superscripts are significantly different (*p* < 0.05, one-way Anova with post hoc Tukey HSD).

**Table 7:** Identified compounds in cheeses obtained by raw milk from dairy cows fed with four diets: CTRL, control diet; L-GS, Lagrein Grape Stalk supplement; CS-GS, Cabernet Sauvignon Grape Stalk supplement; M-GS, Merlot Grape Stalk supplement.

Metabolites Aqueous Fraction (mg/100 g DM)	CTRL	L-GS	CS-GS	M-GS
Acids				
Acetic acid	56.4 ± 13.9 ^AB^	69.0 ± 9.5 ^B^	60.7 ± 20.7 ^AB^	47.8 ± 10.6 ^A^
Citric acid	249 ± 62 ^A^	244 ± 66 ^A^	267 ± 86 ^A^	220 ± 58 ^A^
Formic acid	18.1 ± 6.5 ^A^	23.1 ± 7.9 ^A^	22.8 ± 12.5 ^A^	17.6 ± 2.0 ^A^
Pyruvic acid	11.8 ± 1.9 ^A^	11.9 ± 1.4 ^A^	11.3 ± 2.4 ^A^	10.6 ± 1.6 ^A^
Lactic acid	2716 ± 405 ^A^	2788 ± 335 ^A^	2895 ± 845 ^A^	2474 ± 343 ^A^
Propionic acid	52.4 ± 15.3 ^A^	47.5 ± 20.3 ^A^	54.7 ± 16.2 ^A^	47.7 ± 32.3 ^A^
Aminoacids				
Glycine	7.7 ± 1.6 ^A^	6.9 ± 1.1 ^A^	7.3 ± 1.8 ^A^	6.9 ± 1.9 ^A^
Methionine	18.9 ± 6.5 ^A^	19.9 ± 8.9 ^A^	18.8 ± 8.1 ^A^	16.0 ± 8.3 ^A^
GABA	66.2 ± 21.7 ^A^	73.6 ± 30.5 ^A^	65.1 ± 25.7 ^A^	55.6 ± 29.4 ^A^
Proline	18.7 ± 3.2 ^A^	17.8 ± 2.0 ^A^	18.0 ± 3.8 ^A^	18.4 ± 5.8 ^A^
Tyrosine	18.5 ± 6.1 ^A^	19.6 ± 8.4 ^A^	17.5 ± 6.9 ^A^	18.2 ± 8.4 ^A^
Serine	161 ± 43 ^A^	142 ± 48 ^A^	144 ± 38 ^A^	147 ± 60 ^A^
Valine	41.7 ± 14.3 ^A^	46.4 ± 19.6 ^A^	42.8 ± 20.2 ^A^	35.4 ± 15.2 ^A^
Sugars				
Glucose	38.2 ± 17.2 ^B^	21.5 ± 3.7 ^A^	27.6 ± 17.7 ^AB^	34.8 ± 16.6 ^AB^
Galactose	112.0 ± 45.3 ^A^	109.2 ± 48.0 ^A^	108.6 ± 39.5 ^A^	80.4 ± 20.1 ^A^
Others				
2,3-Butanediol	18.5 ± 6.1 ^A^	19.6 ± 8.4 ^A^	17.5 ± 6.9 ^A^	18.2 ± 8.4 ^A^
Betaine	9.6 ± 2.9 ^A^	9.2 ± 3.2 ^A^	9.1 ± 3.8 ^A^	9.9 ± 3.4 ^A^
Ethanol	19.8 ± 5.4 ^AB^	21.6 ± 3.7 ^B^	20.8 ± 8.2 ^AB^	15.9 ± 4.9 ^A^
Choline	6.58 ± 1.58 ^B^	7.22 ± 0.85 ^B^	6.45 ± 0.79 ^B^	4.82 ± 1.33 ^A^
Glycerol	79.8 ± 26.1 ^B^	53.3 ± 9.9 ^A^	64.7 ± 16.1 ^AB^	69.7 ± 38.5 ^AB^
**Metabolites Lipidic** **fraction (%)**	**CTRL**	**L-GS**	**CS-GS**	**M-GS**
Unsaturated Fatty Acids (UFA)	24.6	24.1	24.7	25.4
Saturated Fatty Acids (SFA)	75.4	75.9	75.3	74.6
18:3 (alpha-linolenic)	1.2	1.1	1.2	1.2
18:2 (linoleic acid)	1.7	1.6	1.7	1.8
1,2-Diacylglycerols (DAG)	0.9	0.8	0.9	0.9
Triacylglycerols (TAG)	33.4	33.7	33.3	33.3
MUFA	21.8	21.4	21.8	22.4
CLA	2.8	2.7	2.9	3.0

For each row, values with A and B superscripts are significantly different (*p* < 0.05, one-way Anova with post hoc Tukey HSD).

**Table 8:** Identified volatile organic compounds (VOCs) in CTRL and cheeses obtained by raw milk from dairy cows fed with four diets: CTRL, control diet; L-GS, Lagrein Grape Stalk supplement; CS-GS, Cabernet Sauvignon Grape Stalk supplement; M-GS, Merlot Grape Stalk supplement.

VOCs (mg/Kg)	CTRL	L-GS	CS-GS	M-GS
Aldehydes				
hexanal	0.21 ± 0.08 ^B^	0.16 ± 0.07 ^A^	0.05 ± 0.05 ^A^	0.25 ± 0.09 ^B^
2-methyl butanal	0.06 ± 0.02 ^A^	0.06 ± 0.01 ^A^	0.05 ± 0.01 ^A^	0.06 ± 0.02 ^A^
3-methyl butanal	0.09 ± 0.03 ^A^	0.11 ± 0.01 ^A^	0.09 ± 0.04 ^A^	0.09 ± 0.03 ^A^
Ketones				
2-butanone	0.23 ± 0.12 ^A^	0.24 ± 0.06 ^A^	0.17 ± 0.07 ^A^	0.20 ± 0.06 ^A^
2-pentanone	0.85 ± 0.27 ^A^	1.50 ± 0.64 ^A^	0.84 ± 0.12 ^A^	1.03 ± 0.66 ^A^
2-heptanone	0.40 ± 0.36 ^A^	0.33 ± 0.25 ^A^	0.21 ± 0.07 ^A^	0.44 ± 0.40 ^A^
acetoin	0.45 ± 0.18 ^B^	0.33 ± 0.05 ^A^	0.22 ± 0.09 ^A^	0.54 ± 0.24 ^A^
2-nonanone	0.07 ± 0.07 ^A^	0.05 ± 0.02 ^A^	0.03 ± 0.02 ^A^	0.07 ± 0.08 ^A^
Alcohols				
Ethanol *	0.53 ± 0.05	0.59 ± 0.05	0.44 ± 0.05	0.30 ± 0.05
2-methyl-1-butanol	0.05 ± 0.02 ^A^	0.06 ± 0.02 ^A^	0.04 ± 0.03 ^A^	0.04 ± 0.02 ^A^
3-methyl-1-butanol	0.20 ± 0.17 ^A^	0.18 ± 0.05 ^A^	0.16 ± 0.06 ^A^	0.10 ± 0.02 ^A^
1-pentanol	0.04 ± 0.01 ^A^	0.04 ± 0.01 ^A^	0.04 ± 0.01 ^A^	0.04 ± 0.02 ^A^
2-ethyl-1-hexanol	0.36 ± 0.20 ^A^	0.31 ± 0.06 ^A^	0.24 ± 0.05 ^A^	0.33 ± 0.06 ^A^
phenylethyl alcohol	0.03 ± 0.01 ^A^	0.02 ± 0.05 ^A^	0.01 ± 0.05 ^A^	0.01 ± 0.05 ^A^
2-heptanol	0.04 ± 0.05 ^A^	0.04 ± 0.03 ^A^	0.02 ± 0.02 ^A^	0.01 ± 0.01 ^A^
1-hexanol	0.08 ± 0.04 ^A^	0.12 ± 0.06 ^A^	0.08 ± 0.02 ^A^	0.09 ± 0.04 ^A^
Esters				
ethyl hexanoate	0.24 ± 0.16 ^B^	0.12 ± 0.041 ^A^	0.14 ± 0.07 ^A^	0.19 ± 0.21 ^A^
ethyl octanoate	0.02 ± 0.02 ^B^	0.01 ± 0.003 ^A^	0.02 ± 0.01 ^A^	0.02 ± 0.02 ^A^
ethyl decanoate	0.02 ± 0.01 ^B^	0.01 ± 0.004 ^A^	0.02 ± 0.01 ^A^	0.01 ± 0.01 ^A^
butyrolactone	0.05 ± 0.04 ^A^	0.04 ± 0.02 ^A^	0.04 ± 0.01 ^A^	0.06 ± 0.03 ^A^
delta-decalactone	0.03 ± 0.01 ^A^	0.04 ± 0.03 ^A^	0.03 ± 0.01 ^A^	0.04 ± 0.02 ^A^
Acids				
acetic acid	2.01 ± 0.63 ^A^	2.05 ± 0.47 ^A^	1.8 ± 0.65 ^A^	1.85 ± 0.66 ^A^
formic acid	0.06 ± 0.08 ^A^	0.04 ± 0.04 ^A^	0.03 ± 0.01 ^A^	0.05 ± 0.03 ^A^
butanoic acid	5.40 ± 2.62 ^B^	1.88 ± 0.76 ^A^	2.59 ± 0.68 ^A^	1.88 ± 0.60 ^A^
pentanoic acid	0.04 ± 0.032 ^B^	0.01 ± 0.01 ^A^	0.03 ± 0.01 ^A^	0.05 ± 0.06 ^A^
hexanoic acid	7.07 ± 3.98 ^B^	2.42 ± 0.92 ^A^	6.61 ± 4.77 ^A^	2.61 ± 0.77 ^A^
heptanoic acid	0.05 ± 0.01 ^B^	0.02 ± 0.02 ^A^	0.05 ± 0.01 ^A^	0.08 ± 0.01 ^B^
octanoic acid	1.70 ± 1.10 ^B^	0.57 ± 0.15 ^A^	1.87 ± 1.29 ^A^	0.69 ± 0.25 ^A^
nonanoic acid	0.07 ± 0.051 ^A^	0.06 ± 0.06 ^A^	0.04 ± 0.02 ^A^	0.10 ± 0.07 ^A^
decanoic acid	0.14 ± 0.09 ^B^	0.06 ± 0.02 ^A^	0.16 ± 0.12 ^A^	0.19 ± 0.20 ^A^

Different superscripts within a row indicate significant differences among diets (*p* < 0.05, one-way Anova with post hoc Tukey HSD). VOCs < 0.01 ppm have not been shown. * Probable environmental contamination during the cheese sampling because ethanol was used for sterilizing the cutter.

## Data Availability

Upon reasonable request, the datasets of this study can be made available from the corresponding author.

## Abbreviations

The following abbreviations are used in this manuscript:

GS	Grape stalk
L-GS	Lagrein Grape stalk
CS-GS	Cabernet Sauvignon Grape stalk
M-GS	Merlot Grape stalk
CTRL	Control diet
NMR	Nuclear magnetic resonance spectroscopy analysis
VOCs	Volatile organic compounds
SCFA	Short-chain fatty acids

## Appendix A

**Table A1:** Chemical composition and inclusion level of concentrate supplements fed to dairy cows DM. Chemical composition was not analytically determined because the hay originated from multiple small plots, which precluded representative sampling for laboratory analysis, and because the same farm-produced hay was intentionally maintained throughout the experimental period to preserve the routine feeding management of the herd.

Parameter	^1^ Concentrate1	^2^ Concentrate2	^3^ Dry Unifeed	^4^ Hay
Inclusion (kg/day, as fed)	7.0	2.0	3.0	12.87
^5^ Dry matter (g/kg as fed)	880	880	875	nd^6^
Crude protein (g/kg DM)	216	109	142	nd^6^
Ether extract (g/kg DM)	32	40	43	nd^6^
Crude fiber (g/kg DM)	78	151	174	nd^6^
Ash (g/kg DM)	77	64	66	nd^6^
Net energy for lactation (MJ/kg DM)	8.2	7.5	6.8	nd^6^
Total dry matter intake (kg/day/cow)	6.16	1.76	2.63	11.45

^1^ Concentrate 1 (Optiplus-19, Consorzio Agrario di Bolzano, Bolzano, Italy); ^2^ Concentrate 2 (En Mix Maiscobs-Kleie, Consorzio Agrario di Bolzano, Bolzano, Italy); ^3^ Dry UNIFEED (Dry UNIFEED for dairy cow; Beikircher Grünland Srl, Lana, Italy). ^4^ Hay dry matter content was assumed based on standard values for hay of similar botanical composition (89% DM). ^5^ Dry matter assumed to be 88% for concentrates with undeclared moisture content. nd^6^:not determined.

**Table A2:** Grape stalks Total polyphenols contet (TPC) expressed as mg catechin equivalents per kg of GS dry matter and elemental composition expressed as mg/Kg DM.

	L-GS	CS-GS	M-GS
TPC	14,621.60	12,771.52	16,531.36
Al	54.36 ± 18.90	55.89 ± 11.97	40.68 ± 0.97
As	<0.03	<0.03	<0.03
B	30.57 ± 0.49	25.66 ± 0.24	28.07 ± 0.08
Ba	31.97 ± 0.21	7.12 ± 0.23	10.96 ± 0.10
Be	<0.03	<0.03	<0.03
Ca	7205.75 ± 69.83	6997.10 ± 17.76	7694.12 ± 219.56
Cd	<0.03	<0.03	<0.03
Co	<0.06	<0.06	<0.06
Cr	0.53 ± 0.01	0.41 ± 0.14	0.27 ± 0.01
Cu	151.55 ± 2.86	226.67 ± 1.45	179.49 ± 1.67
Fe	49.46 ± 7.49	43.97 ± 8.06	34.00 ± 0.04
Hg	<0.06	<0.06	<0.06
K	32,500.84 ± 58.82	46,048.90 ± 168.21	38,040.28 ± 1323.60
Li	0.14 ± 0.01	<0.06	<0.06
Mg	1504.14 ± 5.39	1475.02 ± 27.83	1214.97 ± 33.98
Mn	24.76 ± 0.05	10.75 ± 0.09	29.07 ± 0.13
Mo	<0.1	<0.1	<0.1
Na	40.81 ± 0.06	99.31 ± 1.17	77.27 ± 1.73
Ni	0.91 ± 0.64	0.27 ± 0.08	0.23 ± 0.04
P	2363.88 ± 53.83	5503.91 ± 98.65	3211.610
Pb	0.88 ± 0.01	0.51 ± 0.01	<0.3
S	1145.29 ± 15.89	846.64 ± 7.75	762.32 ± 156.75
Sb	<0.3	<0.3	<0.3
Se	<0.3	<0.3	<0.3
Sn	0.65 ± 0.06	0.80 ± 0.11	0.53 ± 0.17
Sr	20.04 ± 0.15	17.57 ± 0.36	17.85 ± 0.21
Ti	1.53 ± 0.09	1.71 ± 0.28	1.12 ± 0.01
Tl	<0.3	<0.3	<0.3
V	<0.06	<0.06	<0.06
Zn	18.13 ± 0.93	41.97 ± 1.17	18.56 ± 1.37
